# Improvement of Recalcitrant Folliculitis Decalvans With Tirzepatide: A Case Report

**DOI:** 10.7759/cureus.76267

**Published:** 2024-12-23

**Authors:** Kali Morrissette, Stefan Hansen, Michelle Pavlis, John C Murray

**Affiliations:** 1 Department of Dermatology, Duke University School of Medicine, Durham, USA; 2 Department of Dermatology, Duke University Medical Center, Durham, USA; 3 Department of Dermatology, Durham Veterans Affairs Health Care System, Durham, USA

**Keywords:** folliculitis decalvans, glp-1 receptor agonists, hair regrowth, inflammatory alopecia, tirzepatide

## Abstract

Folliculitis decalvans (FD) is a chronic inflammatory alopecia characterized by painful, scarring lesions and recurrent flares, often complicated by secondary bacterial infections. Despite the use of topical and systemic anti-inflammatory or antimicrobial therapies, FD remains challenging to manage, with limited therapeutic advancements. We report a case of recalcitrant FD in a man in his 40s who experienced significant symptom improvement and hair regrowth following the initiation of tirzepatide for weight management. This case highlights a previously unreported therapeutic benefit of tirzepatide in FD, potentially mediated through its anti-inflammatory and immunomodulatory effects. As glucagon-like peptide 1 (GLP-1) receptor agonists gain recognition for their efficacy in other inflammatory skin conditions, such as psoriasis and hidradenitis suppurativa, this case supports further exploration of their role in managing FD and other refractory dermatologic diseases.

## Introduction

Tirzepatide is a gastric inhibitory polypeptide analogue with dual glucagon-like peptide 1 (GLP-1) receptor activity, primarily used for the treatment of type 2 diabetes mellitus and obesity [1]. While its metabolic benefits are well-documented, its potential anti-inflammatory effects remain underexplored. Folliculitis decalvans (FD) is a chronic inflammatory alopecia marked by recurrent flares and scarring, with pathogenesis involving a complex interplay of immune dysregulation, microbial factors, and chronic inflammation, ultimately leading to permanent hair loss [2-4]. Emerging reports suggest its benefit in inflammatory skin conditions such as psoriasis and hidradenitis suppurativa (HS) [5-7]. Here, we present a case of recalcitrant FD that demonstrated rapid improvement following the initiation of tirzepatide for weight loss management, highlighting its potential as a novel therapeutic option for this challenging condition.

## Case presentation

A man in his 40s presented with a 30-year history of FD, with frequent relapses causing pain, bleeding, and drainage that severely affected his quality of life. Multiple prior biopsies revealed a consistent histopathological pattern of severe granulomatous folliculitis and perifolliculitis with marked scarring. Cultures from the affected area were positive for methicillin-sensitive *Staphylococcus aureus*.

Prior ineffective treatments included topical steroids, topical retinoids, calcipotriene cream, crisaborole ointment, ruxolitinib cream, ketoconazole shampoo, benzoyl peroxide wash, oral antibiotics (minocycline, doxycycline, rifampin, clindamycin), hydroxychloroquine, and colchicine. Cryotherapy and intralesional corticosteroid injections were also ineffective.

At the time of starting tirzepatide, the patient was managing FD with clobetasol spray and crisaborole ointment applied every five to seven days. In May 2023, his primary care physician prescribed weekly subcutaneous tirzepatide injections for chronic weight management, starting at 2.5 mg and titrated to 12.5 mg. Over the next nine months, he experienced substantial weight loss of over 50 pounds.

**Figure 1:**
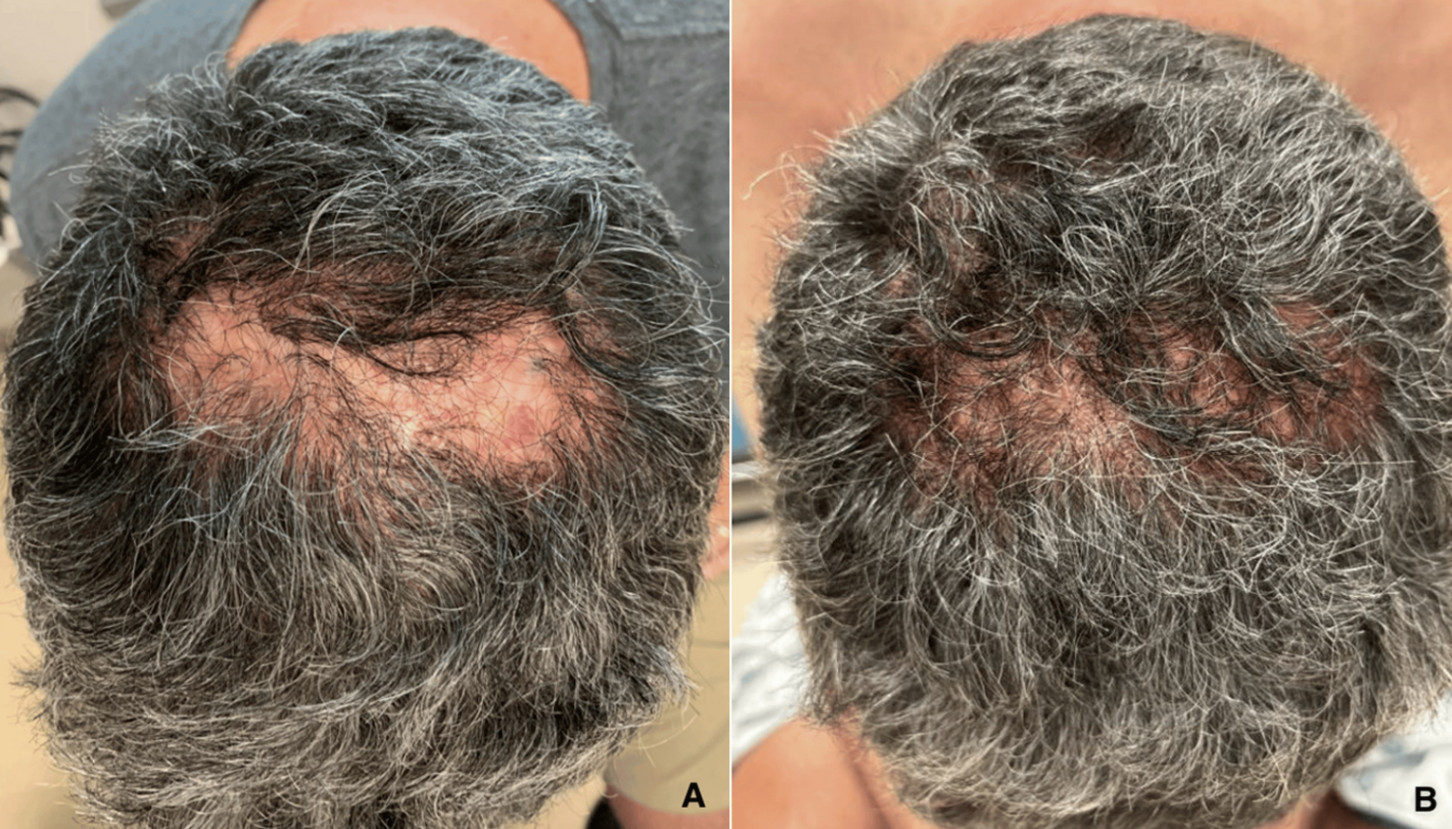
Hair regrowth observed on 7/13/2023 (A) and 2/23/2024 (B), following the initiation of tirzepatide.

Remarkably, during this period, the patient noted significant, unexpected improvements in his FD symptoms, despite no changes to his existing FD treatment regimen. These improvements included substantial reductions in pain, drainage, and flares, with a notable decrease in erythema and fluctuance on physical exam. Additionally, the patient experienced prominent hair regrowth in the affected area (Figure 1).

In May 2024, the patient’s tirzepatide dose was tapered, leading to a flare in FD symptoms. His condition has since stabilized on his current dosage of 7.5 mg weekly, which he continues to maintain.

## Discussion

FD develops through a two-stage process, beginning with disrupted immune responses that lead to bacterial overgrowth and subsequent hair follicle damage [2]. A key feature is the decreased production of essential immune signals, including interleukin-10 (IL-10), tumor necrosis factor-alpha (TNF-α), and IL-6, which perpetuates a chronic cycle of inflammation and bacterial imbalance [2,3]. This underlying pathogenesis highlights the potential for therapeutic interventions targeting both immune dysfunction and microbial imbalance.

GLP-1 agonists have emerged as potential therapeutic agents for inflammatory skin diseases, with promising evidence in conditions like psoriasis and HS [5-7]. In psoriasis, these agents have been associated with significant reductions in Psoriasis Area and Severity Index (PASI) scores, independent of weight loss, by reducing cytokine expression (e.g., TNF-α) and potentially regulating immune cell activity, including peripheral regulatory T-cells [4]. Similarly, in HS, GLP-1 agonists have been linked to reductions in inflammatory markers and improved quality of life, suggesting benefits beyond weight modulation. Given FD's pathogenesis, GLP-1 agonists could address multiple aspects of the disease, including normalizing impaired cytokine production and restoring proper immune responses to follicular microbiota [8].

Despite these promising findings, there are no reported cases of GLP-1 agonists being used to treat FD. Our patient's significant improvement after the initiation of tirzepatide suggests that GLP-1 agonists could represent a new approach to managing FD. By reducing pro-inflammatory cytokines and modulating immune responses, these agents may impact the pathways involved in FD, much like their effects in psoriasis.

Interestingly, while some reports link GLP-1 agonists to hair loss, the relationship between these medications and hair health remains unclear [9]. A recent case report described significant hair regrowth in a patient with androgenic alopecia treated with tirzepatide for insulin resistance, suggesting that improved insulin sensitivity might promote hair growth [10]. In our patient, notable hair regrowth accompanied the improvement in FD, further highlighting the potential role of GLP-1 agonists in supporting hair follicle health.

## Conclusions

We presented the case of a man in his 40s with a 30-year history of recalcitrant FD who experienced remarkable improvement in symptoms and hair regrowth, following the initiation of tirzepatide for weight management. This case highlights the potential anti-inflammatory and immunomodulatory properties of GLP-1 receptor agonists in managing FD, a chronic and refractory dermatologic condition.

The significant clinical improvements observed in this patient underscore the need for further investigation into the therapeutic role of GLP-1 receptor agonists in inflammatory skin diseases, particularly FD. As these agents gain increasing recognition for their efficacy in conditions such as psoriasis and HS, this case broadens their potential application to include FD. Additionally, this report suggests that tirzepatide may provide benefits beyond its metabolic effects, warranting further exploration of its impact on hair health and inflammatory processes.
